# The Role of Family Functioning in the Relationship Between Job Burnout and Parental Burnout in Working Parents: A Moderation Model

**DOI:** 10.1111/jmft.70139

**Published:** 2026-04-27

**Authors:** Daniela Marchetti, Lilybeth Fontanesi, Serena Di Giandomenico, Antonio Pio Facchino, Maria Cristina Verrocchio

**Affiliations:** ^1^ Department of Psychology G. d'Annunzio University of Chieti‐Pescara Chieti Italy

**Keywords:** balanced family functioning, circumplex model, job burnout, parental burnout

## Abstract

Research has underscored the interconnection between work and family domains, showing that an imbalance between demands and resources can lead to job and parental burnout. Our study examined the moderating role of family functioning in the association between job burnout and parental burnout among working parents in Italy. Drawing on Olson's Circumplex Model, balanced family functioning was conceptualized as optimal levels of cohesion and flexibility, reflecting a well‐regulated family dynamic. Three‐hundred‐nine working parents of children aged 0–18 completed a survey assessing sociodemographic characteristics, job burnout, parental burnout, and family functioning. Results indicate that parental burnout was positively related to job burnout and negatively to family functioning. Simple slope analyses showed that the association between job and parental burnout progressively weakened as family functioning increased and became non‐significant beyond a specific threshold. Our findings highlight the clinical value of balanced family functioning's buffering effect on work–family stress processes.

## 1 Introduction

In recent decades, rapid socio‐cultural, economic, and technological changes have profoundly reshaped family dynamics and parenting practices. As expectations of parents rise and the challenge of balancing work and family responsibilities becomes more complex, many parents find themselves under overwhelming stress (Nguyen et al. 2025; Obrenovic et al. 2020).

Within this context, parental burnout has emerged as a significant and growing social concern. A recent study (Roskam et al. 2021) conducted in 42 countries worldwide found that approximately 5% of parents experience parental burnout, rising to 9% in Western countries. It is reasonable to assume that the COVID‐19 pandemic may have increased parental burnout due to social isolation, diminished social support, and managing remote work, homeschooling, and childcare (Achterberg et al. 2021; Freisthler et al. 2022; Racine et al. 2022; Westrupp et al. 2023).

Parental burnout has been conceptualized as a psychological syndrome defined as a “prolonged response to chronic and overwhelming parental stress” (Mikolajczak and Roskam 2018; Roskam et al. 2017). It results from a persistent imbalance between parental demands and available resources, leading to depletion of the latter when protective factors are insufficient to meet the demands (Mikolajczak and Roskam 2018). Parental burnout is characterized by three key dimensions: exhaustion in caring for children, emotional distancing from children, and a sense of low personal accomplishment in parenting. Specifically, emotional exhaustion refers to the sensation of emptiness and overwhelm in one's parental role, resulting in extreme fatigue in fulfilling parental responsibilities and a sense of being drained of energy (Gillis and Roskam 2019; Roskam et al. 2017). Emotional distancing describes the feeling of being less and less involved in child caring, with interactions limited to functional tasks to the detriment of emotional aspects. Finally, lack of parental accomplishment reflects feelings of incompetence and failure in the parental role, along with a perceived inability to achieve meaningful outcomes (Roskam et al. 2017).

As evident in previous studies, parental burnout poses severe risks. It can lead to symptoms of depression, suicidal thoughts, substance abuse, sleep disorders, and marital conflict in parents (Kawamoto et al. 2018; Mikolajczak et al. 2018, 2019). Additionally, parental burnout can result in neglect and aggressive behavior towards children (Mikolajczak et al. 2021), leading to negative consequences for their mental health, including symptoms of depression and anxiety, as well as adverse effects on academic performance (An et al. 2022). Given the significant negative consequences of parental burnout for both parents and children, attention must be paid to the various interrelated factors that may contribute to its onset, maintenance, or reduction.

Among the factors associated with parental burnout, attention has been given to job burnout (Wang et al. 2022, 2023). Job burnout is conceptualized as a psychological syndrome caused by protracted exposure to work‐related stress (Maslach 1993; Maslach and Jackson 1981), and encompasses three key dimensions: overwhelming exhaustion, depersonalization of the beneficiaries of one's work, and a sense of ineffectiveness and lack of accomplishment (Maslach 1993; Maslach and Jackson 1981; Roskam et al. 2017). The exhaustion component represents the core dimension of burnout and refers to the feelings of being overextended and depleted of one's emotional and physical resources. The depersonalization component pertains to a negative, callous, or excessively detached response to various aspects of the job, including the beneficiaries of one's work. The component of reduced efficacy or accomplishment describes the feelings of incompetence and a lack of achievement and productivity at work (Maslach et al. 2001).

Literature emphasizes that work and family are two interconnected domains, with parental burnout and job burnout exerting a bidirectional and mutually reinforcing negative impact on each other (Wang et al. 2022, 2023). Consistent with Conservation of Resources theory (COR; Hobfoll 1989) and spillover models (family‐to‐work and work‐to‐family; Kinnunen et al. 2006), Wang et al. (2023) suggested that excessive investment of personal resources in the work domain may leave parents with insufficient resources to manage family‐related demands, thereby increasing the risk of parental burnout. In this sense, exhaustion and resource depletion at work may spill over into the family domain, undermining parents' capacity to cope with parenting pressures. Particularly in dual‐earner families, the need to manage both professional and parenting responsibilities can challenge family functioning, potentially leading to increased stress levels and a heightened risk of both parental and job burnout (Wang et al. 2022).

Since the family is where parenting takes place (Sekułowicz et al. 2022), an important aspect that influences parental burnout is family functioning (Duarte et al. 2005; Durtschi et al. 2017; Lindström et al. 2011; Lisboa‐Lima et al. 2025; Lu et al. 2024). Following the Circumplex Model of Marital and Family Systems (Olson et al. 1979), families can be assessed based on two fundamental principles: cohesion and flexibility. Cohesion delineates the emotional connectedness among family members. Family flexibility encompasses the effectiveness and implementation of leadership, organization, roles, and relationship guidelines within the family dynamic. Family functioning is thus conceptualized as a dynamic interplay between cohesion and flexibility, with optimal levels of both dimensions being associated with healthier relational patterns and overall family well‐being, while unbalanced levels (either very low or very high) are associated with problematic outcomes (Olson et al. 1979). The effort to maintain this balance becomes even more critical for working families.

**Figure 1:**
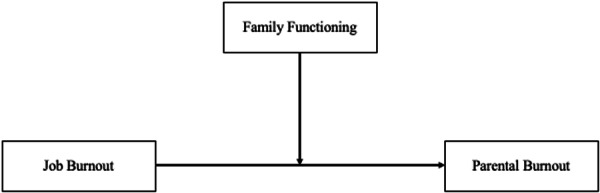
Conceptual moderation model of family functioning (Total Ratio Score) on the association between job burnout and parental burnout. The model was tested controlling for parents' age, gender, educational level, socio‐economic status, and age of the youngest child.

Family functioning plays a crucial role in parental burnout by increasing or decreasing parental demands and the resources required for parenting (Lindström et al. 2011). Several studies found that higher family functioning was associated with lower parental burnout, and lower family functioning was associated with higher levels of parental burnout (Mikolajczak et al. 2018; Pereira et al. 2024; Sekułowicz et al. 2022; Zhang and Zhao 2024). As suggested by Lindström et al. (2011), supportive partner relationships characterized by nurturing interactions, effective communication, and satisfaction with one's partner are associated with lower levels of parental burnout. Agreement between partners on child‐rearing goals and practices, collaborative decision‐making, and mutual recognition of each other's parenting role may further reduce parental burnout, since it is related to lower parenting stress (Durtschi et al. 2017). Conversely, disruptions in family cohesion and flexibility—such as chaotic home environments and the absence of consistent routines—may erode protective family resources (Dumas et al. 2005) and heighten parents' susceptibility to burnout (Mikolajczak et al. 2018). Given evidence that job burnout may spill over into the family domain and increase vulnerability to parental burnout, and that family functioning can either amplify or buffer parenting‐related stress, it is reasonable to assume that family functioning could moderate the relationship between job burnout and parental burnout. Therefore, the present study aims to assess whether the relationship between job burnout and parental burnout is moderated by family functioning in terms of balance between cohesion and flexibility (see Figure 1).

## 2 Methods

### 2.1 Sample and Procedure

The data were collected during the period July–September 2021 using an online self‐administered survey. The study was approved by the Ethical Committee of the Department of Psychological, Health and Territorial Sciences at G. d'Annunzio University of Chieti‐Pescara, and all procedures were performed in accordance with the ethical principles for psychological research, following the Declaration of Helsinki and its revisions (World Medical Association 2001) as well as the ethics guidelines of the American Psychological Association (American Psychological Association 2010). Participants were recruited through social media (survey links with instructions shared on Facebook and Instagram) and via snowball sampling conducted through WhatsApp. The participation was anonymous, free, and voluntary, and the survey took about 15–20 min to complete.

The only inclusion criterion was being a working parent with at least one child aged 0–18 years. A total of 309 working parents completed the survey (263 mothers, *M*_age_ = 41.32, SD = 6.92; 46 fathers, *M*_age_ = 47.11, SD = 7.44). The main characteristics of the study sample are presented in Table 1.

**Table 1:** Sociodemographic characteristics of sample (*n* = 309).

	*N* (%)	*M* (SD)
Age of the parents		42.18 (7.29)
Fathers' age		47.11 (7.44)
Mother's age		41.32 (6.92)
Gender		
Male	46 (14.90)	
Female	263 (85.10)	
Educational level		
Middle school	2 (0.60)	
High school	97 (31.40)	
Degree	126 (40.80)	
Postgraduate degree	84 (27.20)	
Socio‐economic status		
Low (0–15,999 €)	27 (8.70)	
Medium–Low (16,000–33,999 €)	121 (39.20)	
Medium–High (34,000–55,000 €)	137 (44.30)	
High (> 55,000 €)	24 (7.80)	
Age of the youngest child		6.93 (5.38)

Abbreviation: SD, standard deviation.

### 2.2 Measures

#### 2.2.1 Demographic Variables

Drawing from previous studies on parental burnout (Kawamoto et al. 2018; Roskam et al. 2017; Van Bakel et al. 2018), participants were asked about age, gender, level of education, socioeconomic status according to the nationally established brackets, and the age of the youngest child.

#### 2.2.2 Parental Burnout

The Parental Burnout Inventory (PBI; Roskam et al. 2017) is a self‐report questionnaire including 22 items with a 7‐point Likert scale (0 = never; 6 = every day). It consists of three sub‐scales: emotional exhaustion, emotional distancing, and lack of parental accomplishment. A global parental burnout score is calculated by summing the item scores across all dimensions, with higher scores indicating greater levels of parental burnout. As suggested by Roskam et al. (2018), the Parental Burnout Inventory is recommended in research aiming to compare burnout across work and family, utilizing a widely recognized framework.

As the Italian version of the PBI had not been published at the time of the study, a back‐translation procedure was conducted to ensure the linguistic and conceptual equivalence of the instrument. The original version was first translated into Italian by a bilingual expert and subsequently retranslated into English by an independent translator blind to the original version. Discrepancies were discussed and resolved by the research team to achieve semantic consistency. Cronbach's alphas for emotional distancing, emotional exhaustion, and lack of parental accomplishment were 0.83, 0.90, and 0.83, respectively. Overall, PBI alpha was 0.89. These results represent good indicators of internal consistency (Bartholomew and Horowitz 1991; Taber 2018).

#### 2.2.3 Job Burnout

Job burnout was assessed using the Italian version of the Maslach Burnout Inventory (MBI; Maslach and Jackson 1981; Sirigatti et al. 1988), a 22‐item self‐report questionnaire, scored on a 7‐point Likert scale, that examines three dimensions: emotional exhaustion, depersonalization, and personal accomplishment, which consists of reverse‐scored items. A global job burnout score is calculated by summing the item scores across all dimensions, with higher scores indicating greater levels of job burnout. The Cronbach's alpha of the total scale was found to be 0.90.

#### 2.2.4 Family Functioning

Family functioning was measured using the Italian version of the Family Adaptability and Cohesion Evaluation Scale (FACES IV; Loriedo et al. 2013; Olson 2011), a self‐report instrument designed to measure family cohesion and flexibility, which are the primary dimensions of the Circumplex Model of Marital and Family Systems. In the present study, an abbreviated version of 42 items was used, with respondents rating their level of agreement or disagreement using a 5‐point Likert scale (ranging from 1 = strongly disagree to 5 = strongly agree). The questionnaire includes two balanced subscales (cohesion and flexibility) and four unbalanced subscales (disengaged, enmeshed, rigid, and chaotic). The Cronbach's alpha coefficients were 0.81 for the cohesion subscale, 0.73 for the flexibility subscale, 0.64 for the disengaged subscale, 0.60 for the enmeshed subscale, 0.59 for the rigid subscale, and 0.56 for the chaotic subscale.

The standard scoring procedure involves the calculation of ratio scores for both cohesion and flexibility dimensions (Paone et al. 2024), which in turn allows for the calculation of the Total Ratio Score (TRS). The TRS is an index of overall family balance. The ratio scores were calculated using the following formulas (Olson 2011):

1. Cohesion Ratio Score = Balanced Cohesion/[(Disengaged + Enmeshed)/2];
2. Flexibility Ratio Score = Balanced Flexibility/[(Rigid + Chaotic)/2];
3. Total Ratio Score = (Cohesion Ratio Score + Flexibility Ratio Score)/2.

The TRS was used as the primary indicator of family functioning since it provides a theoretically meaningful index of systemic balance within the Circumplex Model, directly reflecting the model's core hypothesis (Olson 2011) that healthier family systems are balanced (TRS > 1), whereas more problematic systems are unbalanced (TRS < 1). Given the modest internal consistency of several subscales of the FACES IV in the present sample, we additionally examined the reliability of the TRS as a derived composite index using a Monte Carlo simulation approach (He 2009; Kelley and Pornprasertmanit 2016). This analysis supported the reliability of the TRS in the present sample (estimated reliability = 0.76, 95% CI [0.65–0.88]).

### 2.3 Data Analyses

SPSS version 26 was utilized to execute the reliability analysis, the correlation analysis between the variables, and the moderation model analysis using the PROCESS extension (Hayes 2022). Cases with missing data on the study variables were handled using pairwise deletion (available cases analysis), such that each analysis was based on all cases with available data for the variables involved. The reliability of the scales was assessed using the Cronbach α coefficient. Values above 0.70 are generally considered acceptable, indicating adequate reliability. Values between 0.80 and 0.90 are regarded as good, while values above 0.90 may suggest excellent internal consistency (Taber 2018). For the derived composite ratio score, reliability was estimated via Monte Carlo simulation based on classical test theory and composite‐score reliability methods (He 2009; Kelley and Pornprasertmanit 2016) using 5000 replications (random seed = 12,345), as Cronbach's *α* is not directly applicable to ratio indices. Reliability values ≥ 0.70 were considered adequate (DeVellis 2016; Nunnally and Bernstein 1994).

Descriptive statistics and correlation analyses were conducted to examine associations among parental burnout, job burnout, family functioning, and relevant sociodemographic variables. Pearson's *r* coefficients were used for associations among continuous variables, whereas Kendall's τ coefficients were employed for associations involving ordinal variables. Point‐biserial correlation was used for assessing the association between gender and parental burnout.

Moderation analysis was conducted to examine the moderating influence of family functioning on the relationship between job burnout and parental burnout. Covariates were selected based on theoretical relevance and prior literature, including parent gender, age, educational level, socioeconomic status, and age of the youngest child (Giraldo et al. 2022; Mikolajczak et al. 2023; Ren et al. 2024).

Continuous predictors involved in the interaction term were mean‐centered by subtracting the sample mean from each individual score, using the centering option provided by the PROCESS macro. The interaction term was computed as the product of the mean‐centered predictor and moderator. Standardized regression coefficients (β) were obtained by estimating the model using standardized (z‐scored) variables. Significant interactions were probed and plotted using simple slopes at one standard deviation below the mean, the mean, and one standard deviation above the mean of the moderator, including tests of whether each simple slope was significantly different from zero. In addition, the Johnson–Neyman technique was used to identify the value of the moderator at which the association between job burnout and parental burnout transitioned from statistically significant to non‐significant.

## 3 Results

### 3.1 Correlation Between Parental Burnout and Other Variables

As shown in Table 2, a moderate positive correlation between parental burnout and job burnout emerged. Moderate negative correlations were observed between parental burnout and family functioning, as well as between job burnout and family functioning. No significant associations emerged between sociodemographic variables and parental burnout. In particular, point‐biserial correlation analysis revealed that parents' gender was not significantly associated with parental burnout (*r*_pb_ = −0.01, *p* = *ns*). Consistently, Kendall tau analyses showed that parents' educational level (*τ* = −0.05, *p* = *ns*) and socio‐economic status (*τ* = −0.03, *p* = *ns*) were not significantly correlated with parental burnout. Finally, Pearson correlation coefficients indicated that parents' age (*r* = −0.05, *p* = *ns*) and the age of the youngest child (r = −0.07, *p* = *ns*) were not significantly correlated with parental burnout.

**Table 2:** Descriptive statistics and Pearson correlations among the study variables.

	*M*	SD	1	2
1. Parental burnout	33.60	20.50	—	
2. Job burnout	36.90	18.73	0.44**	—
3. Family functioning	0.43	0.10	−0.48**	−0.39**

*Note:* Parental burnout, *N* = 309; Job burnout, *N* = 309; Family functioning, *N* = 281.

Abbreviation: SD, standard deviation.

*p < 0.05

**p < 0.001.

### 3.2 Moderation

To examine the moderating effect of family functioning on the relationship between job burnout and parental burnout, a moderation analysis was performed. Demographic variables, including parent age, gender, educational level, socioeconomic status, and age of the youngest child, were entered as covariates to account for their potential influence on parental burnout, based on prior research and regardless of statistical significance. As depicted in Table 3, job burnout positively predicted parental burnout (*b* = 0.40, *β* = 0.37, *p* < 0.001), indicating that higher levels of job burnout were related to higher levels of parental burnout. Family functioning was also a significant predictor of parental burnout (*b* = –74.26, *β* = −0.38, *p* < 0.001), such that better family functioning was associated with lower parental burnout.

**Table 3:** Moderation analysis, with *Parental Burnout* as dependent variable.

	Parental burnout			
Predictor	*b*	*SE*	*β*	*p*
Job burnout	0.40	1.06	0.37	0.000
Family functioning	−74.26	10.79	−0.38	0.000
Job burnout × Family functioning	−1.06	0.46	−0.10	0.021
Age of the parents	−0.23	0.24	−0.08	0.339
Gender	−5.64	2.98	−0.09	0.059
Educational level	2.73	1.37	0.10	0.046
Socio‐economic status	0.39	1.42	0.01	0.779
Age of the youngest child	−0.10	0.32	−0.03	0.744

Abbreviation: SE, standard error.

**Figure 2:**
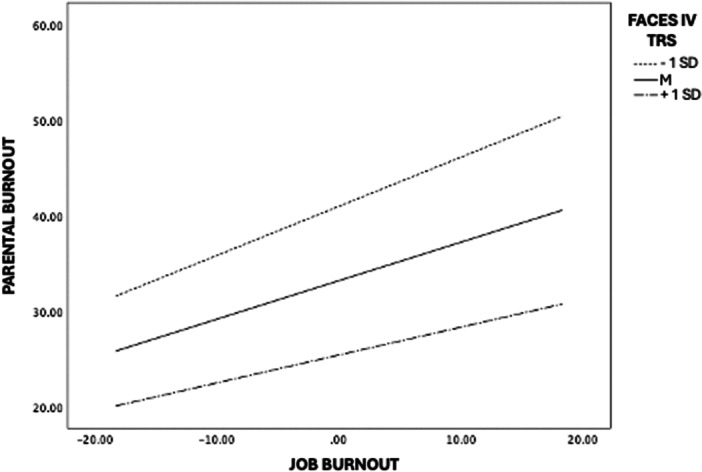
Plots of significant moderation of family functioning on the association between job burnout and parental burnout.

Notably, the interaction between job burnout and family functioning was significant (*b* = –1.06, *β* = −0.10, *p* < 0.05), indicating that family functioning moderated the association between job burnout and parental burnout. As shown in Figure 2, the positive association between job burnout and parental burnout was strongest at lower levels of family functioning and progressively weaker at average and higher levels of family functioning, suggesting a buffering effect of family functioning. Simple slopes analyses indicated that the association between job burnout and parental burnout was significant at low (−1 SD; *b* = 0.51, *β* = 0.47, *p* < 0.001), mean (*b* = 0.40, *β* = 0.37, *p* < 0.001), and high (+1 SD; *b* = 0.29, *β* = 0.27, *p* < 0.001) levels of family functioning, confirming that each slope differed significantly from zero.

Johnson–Neyman analyses further showed that the association between job burnout and parental burnout was statistically significant for values of family functioning below 0.1828, corresponding to 96.1% of the sample. At higher levels of family functioning, the association between job burnout and parental burnout was no longer statistically significant, indicating that high family functioning substantially attenuated the link between job burnout and parental burnout. Among the covariates, educational level was positively associated with parental burnout (*b* = 2.73, *β* = 0.10, *p* < 0.05), whereas the remaining covariates were not significant. Overall, the model accounted for 37% of the variance in parental burnout.

## 4 Discussion

Over the past few years, relevant sociological and demographic changes have occurred, including an increasing prevalence of dual‐income households (Wang et al. 2022). This implies organizational and childcare difficulties (Finstad et al. 2024; Pascucci et al. 2021), which may result in elevated stress levels and an augmented risk of both parental and job burnout. In this context, research on parental burnout has spread (Marchetti et al. 2020; Polizzi et al. 2022; Ren et al. 2024; Yang et al. 2025), but further examination is still necessary to ascertain the relationship between parental burnout and job burnout, especially in Western countries (Wang et al. 2022, 2023).

The present study contributes to this literature by examining the interplay between job burnout and parental burnout, with a specific focus on the moderating role of family functioning. Consistent with previous research suggesting a bidirectional relationship between the two forms of burnout (Wang et al. 2022, 2023), our findings confirm a significant positive association between job and parental burnout. In line with previous literature (Wang et al. 2023), this result can be interpreted within the Conservation of Resources theory (COR; Hobfoll 1989) and spillover models (work‐to‐family; Kinnunen et al. 2006). Specifically, exhaustion and resource depletion at work may reduce parents' available emotional and cognitive resources, leaving them less able to cope with parenting demands. As work‐related strain transfers into the family domain, parents may experience diminished capacity to manage parenting stressors, increasing their vulnerability to parental burnout (Wang et al. 2023). This mechanism may be particularly pronounced in dual‐earner families, where balancing work and childcare responsibilities places additional pressure on family functioning (Wang et al. 2022). Similarly, this finding aligns with prior research highlighting the role of cultural context in shaping spillover processes between job burnout and parental burnout (Wang et al. 2022, 2023). Our Italian results support the relevance of work‐to‐family spillover pathways, consistent with evidence that work may be prioritized among some age groups in Italy (i.e., 59% of individuals aged 30–49; Pew Research Center 2021).

However, the key contribution of this study lies in demonstrating that family functioning, when balanced in terms of cohesion and flexibility, significantly buffers the impact of job burnout on parental burnout. This result gains particular relevance in the context of contemporary society, where job demands, job insecurity, and work‐family conflict have a significant impact on individuals' lives (Pascucci et al. 2021). This moderation effect aligns with the Circumplex Model of Marital and Family Systems (Olson 2011; Olson et al. 1979), which posits that adaptive family functioning is characterized by optimal levels of cohesion and flexibility. When these dimensions are balanced, families are more capable of managing external stressors, such as work‐related demands, without these pressures translating into parental distress. Our results suggest that families with higher levels of functioning may be better equipped to absorb the effects of occupational stress, thereby protecting parents from developing symptoms of burnout in the parenting domain.

This pattern may also help explain why the association between job burnout and parental burnout became non‐significant once TRS exceeded 0.1828. Conceptually, the Circumplex Model posits that families with more balanced cohesion and flexibility are better able to respond adaptively to external stressors (Olson 2011; Olson et al. 1979). When family functioning reaches sufficiently high levels, relational and organizational resources, such as emotional support, effective communication, shared responsibility, and flexible routines, may protect parents from carrying work‐related exhaustion into the parenting domain (Durtschi et al. 2017). From a resource‐based perspective, such family‐system resources may offset depletion originating from the work context, thereby disrupting the work‐to‐family spillover process (Kinnunen et al. 2006). In contrast, when TRS values are below this threshold, family functioning may be generally positive but still characterized by vulnerabilities that reduce its buffering capacity, allowing job‐related strain to continue translating into parental burnout. Importantly, the observed threshold should be interpreted cautiously and warrants replication in future studies.

Interestingly, among the covariates, educational level was the only variable significantly associated with parental burnout, with parents with higher levels of education reporting higher levels of parental burnout. One possible explanation is that more highly educated parents may hold particularly high expectations for their children and endorse stronger perfectionistic standards, which are factors associated with parental burnout (Mikolajczak et al. 2023; Wu et al. 2022). A further hypothesis is that highly educated parents are more likely to hold demanding professional positions, which may increase exposure to work–family conflict. In turn, the combination of elevated work demands and intensive parenting responsibilities may exacerbate the imbalance between parenting‐related demands and available resources, thereby increasing vulnerability to parental burnout (Mikolajczak et al. 2023; Yang et al. 2025). These results underscore the importance of examining parental burnout from a complex and multidimensional perspective, investigating the role of other potential factors that may influence the relationship between job burnout and parental burnout (e.g., work–family conflict, perceived parental support, presence of children with diagnoses, and cultural factors; Ren et al. 2024; Wang et al. 2022). Such an approach is essential for achieving a clearer and more comprehensive understanding of the underlying mechanisms of this relationship.

The present findings highlight important clinical implications. First, parental burnout should not be seen only as an individual difficulty. Instead, it could be a process integrated into work‐family interactions. Therefore, clinicians working with parents who report symptoms of parental burnout may benefit from systematically assessing work demands and the stress these may cause parents. They should also evaluate the balance of family functioning, including the degree of cohesion and flexibility within the family unit. Moreover, framing burnout as a process of resource depletion and spillover can help parents better recognize and validate their partner's difficulties, thereby strengthening mutual understanding and support, improving overall family functioning, and promoting overall parental well‐being. In terms of clinical intervention with couples and families, the results underscore the potential utility of brief, targeted interventions focused on restructuring family routines. Since balanced family functioning helps mitigate the link between job burnout and parental burnout, therapeutic work may strengthen emotional bonding, mutual support, profitably target family problem‐solving skills, boundary renegotiation, adaptive role redistribution, and constructive conflict management. Moreover, enhancing predictability, role clarity, and shared organization within the household may foster greater cohesion and functional stability, thereby reducing vulnerability to stress spillover from the work domain. Finally, the evidence highlights the importance of interventions designed to strengthen systemic flexibility. By promoting balanced levels of cohesion and flexibility, clinicians may enhance the family system's buffering capacity and mitigate the impact of job burnout on parental burnout.

This study has some limitations. First, although the demographic statistics were similar to previous studies (e.g., Kawamoto et al. 2018; Roskam et al. 2017; Van Bakel et al. 2018) that allow some comparison with the Italian language sample, it is important to exercise caution when making generalizations based on this sample, both in a broader context and specifically regarding the Italian population. As in most studies regarding parental burnout (e.g., Sorkkila et al. 2025; Yang et al. 2025), the main limitation is the high prevalence of mothers in the sample. Moreover, the current study used a cross‐sectional design. Several unbalanced FACES IV subscales showed modest internal consistency in the present sample, which may have attenuated associations involving family functioning. To address this concern, we directly evaluated the reliability of the derived Total Ratio Score using a Monte Carlo simulation approach (He 2009; Kelley and Pornprasertmanit 2016), which indicated adequate score precision in the present sample; nonetheless, replication in larger and independent samples is warranted.

These limitations provide useful suggestions for future research. Future studies should prioritize examining factors associated with parental burnout by expanding the knowledge of possible risk and protective factors that could intervene in the relationship between job burnout and parental burnout. Longitudinal studies are also crucial for understanding the evolving associations over time, especially in Western countries. Additionally, future research should examine parental burnout in a larger sample of fathers. Finally, future studies may benefit from examining specific subdimensions of job burnout, parental burnout, and family functioning to determine which components drive the observed effects.

## 5 Conclusion

Work and family are closely interconnected domains, and an ongoing imbalance between demands and resources in both areas can lead to both job and parental burnout (Wang et al. 2022, 2023). Since parenting occurs within the family context (Sekułowicz et al. 2022), family functioning plays a key role in influencing parental burnout (Duarte et al. 2005; Durtschi et al. 2017; Lindström et al. 2011; Lisboa‐Lima et al. 2025; Lu et al. 2024).

The present study highlighted the significant association between parental burnout, job burnout, and family functioning. In particular, the present data suggest that balanced family functioning mitigates the negative effects of job burnout on parental burnout. Interestingly, above a certain threshold, family functioning showed a buffering effect. These findings have important theoretical and practical implications. Theoretically, they support an ecological and systemic perspective on parental burnout, emphasizing the importance of contextual resources—such as family relationships—in shaping individual outcomes. Practically, they highlight the need for interventions that go beyond individual stress management, promoting relational functioning within families. Prevention strategies and interventions designed to enhance family functioning, implemented at both systemic and individual levels, may serve to alleviate burnout among parents. Mental health support and stress reduction programs could play a pivotal role in this context. Furthermore, recognizing the interrelated nature of job burnout and parental burnout is essential when developing family and work‐related policies. Offering comprehensive support to working parents may contribute to improved family functioning and a reduction in burnout.

## Funding

The authors have nothing to report.

## Data Availability

The data that support the findings of this study are available from the corresponding author upon reasonable request.
